# A chromosomal-scale reference genome of the New World Screwworm, *Cochliomyia hominivorax*

**DOI:** 10.1093/dnares/dsac042

**Published:** 2022-11-12

**Authors:** Sophie Tandonnet, Flavia Krsticevic, Tatiana Basika, Philippos A Papathanos, Tatiana T Torres, Maxwell J Scott

**Affiliations:** Departamento de Genética e Biologia Evolutiva, Instituto de Biociências, Universidade de São Paulo (USP), São Paulo, SP 05508-090, Brazil; Department of Entomology, Robert H. Smith Faculty of Agriculture, Food and Environment, Hebrew University of Jerusalem, Rehovot 7610001, Israel; Department of Entomology and Plant Pathology, North Carolina State University, Campus Box 7613, Raleigh, NC 27695-7613, USA; Department of Entomology, Robert H. Smith Faculty of Agriculture, Food and Environment, Hebrew University of Jerusalem, Rehovot 7610001, Israel; Departamento de Genética e Biologia Evolutiva, Instituto de Biociências, Universidade de São Paulo (USP), São Paulo, SP 05508-090, Brazil; Department of Entomology and Plant Pathology, North Carolina State University, Campus Box 7613, Raleigh, NC 27695-7613, USA

**Keywords:** *Cochliomyia hominivorax*, HiC genome, chromosomal assembly, Calliphoridae, ectoparasite

## Abstract

The New World Screwworm, *Cochliomyia hominivorax* (Calliphoridae), is the most important myiasis-causing species in America. Screwworm myiasis is a zoonosis that can cause severe lesions in livestock, domesticated and wild animals, and occasionally in people. Beyond the sanitary problems associated with this species, these infestations negatively impact economic sectors, such as the cattle industry. Here, we present a chromosome-scale assembly of *C. hominivorax’s* genome, organized in 6 chromosome-length and 515 unplaced scaffolds spanning 534 Mb. There was a clear correspondence between the *D. melanogaster* linkage groups A–E and the chromosomal-scale scaffolds. Chromosome quotient (CQ) analysis identified a single scaffold from the X chromosome that contains most of the orthologs of genes that are on the *D. melanogaster* fourth chromosome (linkage group F or dot chromosome). CQ analysis also identified potential X and Y unplaced scaffolds and genes. Y-linkage for selected regions was confirmed by PCR with male and female DNA. Some of the long chromosome-scale scaffolds include Y-linked sequences, suggesting misassembly of these regions. These resources will provide a basis for future studies aiming at understanding the biology and evolution of this devastating obligate parasite.

## Introduction

The New World Screwworm (NWS), *Cochliomyia hominivorax* (Calliphoridae), is the most important myiasis-causing species in America.^1^ It is an obligate parasite in which the screwworm larvae feed on live tissue in the infested wound of mammals (cutaneous and traumatic myiasis). It is currently found only in South America and some Caribbean islands as it was successfully eradicated from Central and North America using the sterile insect technique or SIT.^2,3^ To prevent its reemergence in Central and North America, sterile flies are mass reared in a facility in Pacora, Panama and released everyday along the Colombian border, which is costly.^3^ Efforts to control its populations in South America heavily rely on the application of insecticides, which is harmful to the environment and is gradually becoming less efficient due to the emergence of resistant populations.^4^

The injuries caused by the larvae are very destructive to the tissues invaded and make the host susceptible to other Dipteran infestations and to bacterial infections.^1,5^ If not treated rapidly, the condition can develop into septicemia and death of the host in the worst cases.^1^ In humans, it mainly affects populations suffering from poor access to good sanitary conditions.^6^

The screwworm myiasis, affecting both humans and animals, is considered a zoonotic disease of importance since it has a huge animal host reservoir (both domestic and wild).^1^ Zoonoses are diseases that can be transmitted from animals to humans. It has been estimated that most emerging diseases of the last century are zoonoses.^7–9^ Zoonotic pathogens and parasites have been at the origin of considerable health problems for both humans and domestic animals.^9^ They also severely impact some economic sectors. In the case of the screwworm, the annual economic losses in South America were estimated to be in the order of USD 3.6 billion.^10^ The presence of screwworm can also present a challenge for wildlife management as shown by the loss of Key deer during the screwworm outbreak in the Florida Keys in 2016.^11^

Recently, the genome of *C. hominivorax* has been sequenced^12,13^ and the CRISPR/Cas9 genome engineering technique has been successfully employed.^14,15^ These fundamental resources are enabling us to further understand the biology of this species, especially the basis of its parasitic lifestyle.

In *C. hominivorax*, the chromosome complement consists of five pairs of approximately equal sized autosomes and a smaller pair of largely heterochromatic sex chromosomes (X and Y).^16^ Here, we present a chromosome-scale assembly of *C. hominivorax*’s genome and the identification of putative genes and genomic regions belonging to the sex chromosomes. This update provides valuable resources for future genetic or evolutionary studies, which could be used to better control *C. hominivorax*’s populations.

## Materials and methods

### Specimen collection and DNA extraction

As described previously,^12^ 6 h mixed sex embryos were collected from an inbred strain of the J06 wild type strain of *C. hominivorax* at the COPEG biosecurity plant in Panama. The embryos were quick-frozen in liquid nitrogen and shipped in a container containing liquid nitrogen to North Carolina. Subsequently, the frozen embryos were shipped on dry ice to Dovetail for HiC library preparation.

For CQ analysis, genomic DNA was prepared from adult males and female flies as previously described.^17^ Illumina DNA library preparation and Illumina DNA sequencing were performed by the Genomic Sciences Laboratory at North Carolina State University following standard procedures.

### Chicago library preparation and sequencing

A Dovetail Chicago library was prepared following established procedures.^18^ Briefly, ~500 ng of high molecular weight gDNA was reconstituted into chromatin in vitro and fixed with formaldehyde. Fixed chromatin was digested with DpnII, the 5ʹ overhangs filled in with biotinylated nucleotides, and then free blunt ends were ligated. After ligation, crosslinks were reversed and the DNA purified from protein. Purified DNA was treated to remove biotin that was not internal to ligated fragments. The DNA was then sheared to ~350 bp mean fragment size and sequencing libraries were generated using NEBNext Ultra enzymes and Illumina-compatible adapters. Biotin-containing fragments were isolated using streptavidin beads before PCR enrichment of each library. The libraries were sequenced on an Illumina HiSeq X to produce 108 million 2 × 150 bp paired end reads.

### Dovetail HiC library preparation and sequencing

A Dovetail HiC library was prepared following established procedures.^19^ Briefly, for each library, chromatin was fixed in place with formaldehyde in the nucleus and then extracted. After chromatin fixation, the same steps (as conducted for the Chicago library preparation) were followed. The libraries were sequenced on an Illumina HiSeq X to produce 143 million 2 × 150 bp paired end reads.

### Scaffolding with Chicago and HiC HiRise

The input de novo assembly, Chicago library reads, and Dovetail HiC library reads were used as input data for HiRise, a software pipeline designed specifically for using proximity ligation data to scaffold genome assemblies.^18^ An iterative analysis was conducted. First, Chicago library sequences were aligned to the draft input assembly using a modified SNAP read mapper (http://snap.cs.berkeley.edu). The separations of Chicago read pairs mapped within draft scaffolds were analyzed by HiRise to produce a likelihood model for genomic distance between read pairs, and the model was used to identify and break putative misjoins, to score prospective joins, and make joins above a threshold. After aligning and scaffolding Chicago data, Dovetail HiC library sequences were aligned and scaffolded following the same method.

This Whole Genome Shotgun project has been deposited at DDBJ/ENA/GenBank under the accession PYHX00000000. The version described in this paper is version PYHX02000000. Additionally the fasta and gff files for this assembly have been deposited at Dryad, dataset doi:10.5061/dryad.d7wm37q4j.

### Post-assembly steps

We used a combination of programs to further annotate the repeats (particularly transposons and retrotransposons) in the genome of *C. hominivorax*: TransposonPSI (http://transposonpsi.sourceforge.net), LTR_harvest,^20^ LTR_Digest,^21^ LTR_finder (version 1.0.7),^22^ RepeatModeler (version 2.0.1) (http://repeatmasker.org/RepeatModeler/)^23^ and RepeatMasker (version 4.0.9-p2) (www.repeatmasker.org/). The genes predicted in our previous assembly using BRAKER^12^ were lifted-over to the current assembly using the pipeline ‘flo’.^24^ The mitochondrial scaffolds were identified by BLASTN (version 2.9.0^25^) using as a reference the complete mitochondrial genome of *C. hominivorax* available on NCBI (Accession number NC_002660.1^26^). The scaffolds identified were analyzed using the webserver MITOS2.^27,28^ All contained the complete mitochondrial genome with some region duplicated. We manually removed the duplicated part from the best quality scaffold and oriented it as the reference mitochondrial genome. The curated mitochondrial genome and its annotation was included in the genome file and annotation file, respectively. We used BUSCO version 5.beta.1^29^ to assess genome and proteome completeness.

### Macro-synteny and gene order analyses between *C. hominivorax* and *Drosophila melanogaster*

To examine the overall macro-synteny between *C. hominivorax* and *D. melanogaster*, we used the location of orthogroups identified using BUSCO, version 5.beta.1.^29^ We visualized the macro-synteny pattern using ggplot2^30^ from R. Best reciprocal BLAST hits and MCScanX^31^ were used to identify collinear blocks of genes between both species. These were visualized using the Accusyn web server (https://accusyn.usask.ca/).

### Identification of X- and Y-linked genomic regions and genes

We use differential coverage between male and female whole genome sequencing (WGS) libraries to identify putative X and Y regions and genes. The WGS libraries consisted of approximately 224.8 Mb and 202.5 Mb Illumina reads for the female and male libraries respectively.

The Chromosome Quotient (CQ),^32^ i.e. the female/male coverage ratio, was computed for (i) non-overlapping 10,000 bp regions along the genome assembly scaffolds (“region CQ”), (ii) for each gene (“gene CQ”) and (iii) for kmers of 25 bp long. As females are XX and males are XY, we considered CQ values close to zero (CQ < 0.2) suggestive of a Y chromosome assignment and, conversely, CQ values above two as an X chromosome signature.

For the region CQ calculation, the raw female and male reads were trimmed to remove poor quality regions using Skewer, version 0.2.2.^33^ The trimmed reads were aligned to the repeat masked genome using bwa, version 0.7.17-r1188.^34^ We used the tool ‘bamCoverage’ from the deeptools package, version 3.5.1^35^ to determine the normalized coverage (RPKM) in each non-overlapping window of 10,000 bp.

The CQ value for each window was calculated by dividing the female by the male normalized coverages for each window. A region with CQ above 2 was tagged as a potential X chromosome region, while a region with CQ below 0.2 was identified as a potential Y chromosome region. Scaffolds with at least 40% of their length tagged X or Y were considered good sex chromosome candidates. We visualized the male and female coverages along the scaffolds using the Interactive Genome Viewer (IGV).

For X- and Y-linked gene assignment, we used both the gene CQ and the context region CQ (CQ values of the 10 kb region in which the putative X or Y gene is localized). The gene CQ values were calculated by quantifying the female and male reads aligned to the putative genes using the Bowtie2 aligner.^36^ We used the Illumina library sizes to normalize the male and female count numbers, and the ratio between female and male coverages (CQ) was calculated for each gene. Genes were categorized as X (or Y) if the gene CQ, as well as the region CQ, were above two (or below 0.2 for Y genes).

To discover potential Y material on a fine scale (within chromosomal-scale scaffolds), we identified kmers of 25 bp along the scaffolds uniquely present in male WGS data. Adjacent Y-unique kmers were merged into Y-unique regions using the Bedtools ‘merge’ program.^37^ The density of these Y unique regions was plotted along the chromosomal-scale scaffolds in 1.8 Mbp non-overlapping windows with the package ggplot2.

### Validation of putative Y regions and genes

We conducted PCR experiments in adult male and female genomic DNA to validate the Y-linkage of the putative Y genes and regions assigned through the CQ method. Specific primers were designed to span four unplaced scaffolds (Supplementary Table S1) and four potential Y genes (Supplementary Table S2). A total of 50 ng of gDNA was used as a template in a 20 µL PCR reaction with Q5 DNA polymerase. Samples were resolved in 2% agarose gels. For long template PCR, a combination of OneTaq and Q5 DNA polymerase was used, with an extension time of 3 minutes, in a 50 µL PCR reaction. Samples were resolved in 0.7% agarose gels.

## Results and discussion

### Basic statistics and BUSCO scores

The chromosome-scale assembly greatly improved the continuity of the genome (Table 1), resulting in 6 chromosomal length scaffolds, the mitochondrial genome and 515 unplaced scaffolds. The repeats were reannotated using a combination of programs (see Methods) resulting in 44.3% of the genome masked as repetitive. Metrics for each of the chromosomal length scaffolds are shown in Supplementary Table S3. The BUSCO score for genome completeness included 97.4% complete BUSCO Orthogroups using the diptera-odb10 database (Table 2). The assembly size of 534 Mbp is essentially unchanged from the first assembly (Table 1) and is 90 Mbp larger than the size measured by flow cytometry.^38^ As noted previously,^12^ this could indicate that the residual heterozygosity in the inbred strain led to divergent alleles to be assembled onto separate contigs. If so, a trio-binning approach could be used to obtain a haplotype-resolved assembly that could be closer to the measured size.^39^

**Table 1.** Basic statistics of the current version of *C. hominivorax*’s genome compared to *D. melanogaster’*s and the previous *C. hominivorax’*s genome

	*C. hominivorax* (HiC_v2)	*C. hominivorax*	*D. melanogaster* (GCF_000001215.4,Release 6 plus ISO1 MT)
Reference	PRJNA438970	PRJNA438970	PRJNA13812
Span (bp) scaffold >1000bp	534,399,004	534,084,880	143,726,002
Number of Scaffolds	6 chromosomal scaffolds + 1 mitochondrial + 515 unplaced scaffolds	3,663	7 chromosomal entities + 1 mitochondrial + 1,862 unplaced scaffolds
Longest scaffold/contig	123,435,726	5,462,925	32,079,331
Scaffold N50 (bp)	101,522,387	616,429	25,286,936
Proportion masked bases (repeats)	236,881,488 (44%)	241,520,001 bp (45%)	32,060,083 bp (22%)
CDS content	33,734,105	33,102,592	22,425,816
Intron content	125,243,883	121,655,471	40,228,708
# protein-coding genes	21,174	21,228	13,931

**Table 2.** BUSCO scores of *C. hominivorax*’s chromosome-scale genome (using the diptera database, *n* = 3,285)

	Complete	Complete and single	Complete and duplicated	Fragmented	Missing
Genome Busco	97.4%	95.4%	2.0%	0.8%	1.8%
Proteome Busco	96.7%	85.3%	11.4%	1.9%	1.4%

### Macro-synteny and collinearity between *C. hominivorax* and *D. melanogaster*

**Figure 1.**
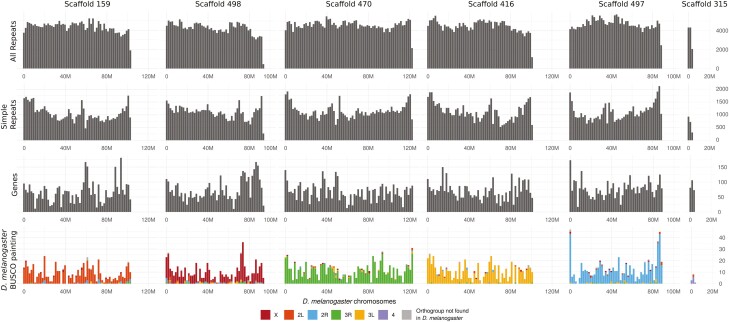
Features along *C. hominivorax*’s chromosome-scale scaffolds. The gene content of each chromosomal entity is largely conserved between *D. melanogaster* and *C. hominivorax* (bottom panel).

There was a clear correspondence between the chromosomal elements of *D. melanogaster* and *C. hominivorax*’s chromosomal-scale scaffolds (Figure 1, bottom panel). The smallest of the six large scaffolds in the *C. hominivorax* assembly, scaffold 315, corresponded primarily to the dot chromosome (chromosome 4) of *D. melanogaster*. The dot chromosome of *D. melanogaster* was previously shown to be an ancient X chromosome in Diptera.^40^ In the related Australian sheep blowfly *Lucilia cuprina*, most of the X-linked genes are orthologs of the dot chromosome genes.^17,41^ Thus, it is likely that scaffold 315 corresponds to the euchromatic part of the X chromosome.

Although the gene content of the chromosomal elements was maintained during the divergence between *D. melanogaster* and *C. hominivorax*, the gene order was poorly conserved (Supplementary Figure S1). Only 7152 (13.52%) of the homologous genes were collinear between both species, within 420 collinear blocks (ranging from 6 to 30 collinear genes). Only one collinear block was found between non-corresponding *D. melanogaster-C. hominivorax* chromosomal elements (Chom_4 and Dmel_3L) (Supplementary Table S4). This reveals that, although genes remain on the same chromosome during the divergence between *D. melanogaster* and *C. hominivorax*, intrachromosomal gene rearrangements have occurred at a faster pace.

### X chromosome scaffold and gene composition

**Figure 2.**
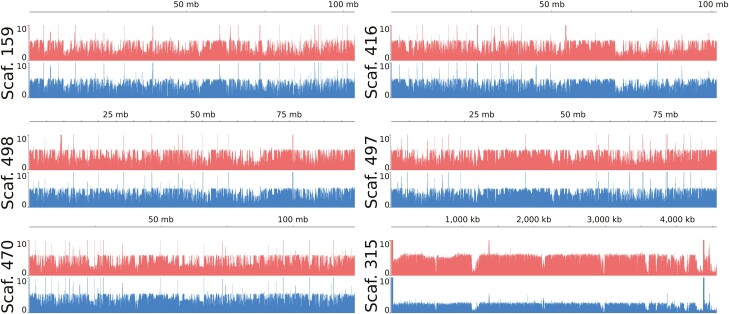
Coverage of female (red) and male (blue) reads across the six chromosomal-scale scaffolds. The male coverage is about half the female coverage for scaffold 315 (bottom-right), an indication that this scaffold is likely from the X chromosome.

Male- and female-specific whole-genome sequencing was used to differentiate potential X and Y regions and scaffolds. Using differential read coverage information of the sex-specific libraries, we calculated the Chromosome Quotient (CQ)^32^ in non-overlapping windows of 10,000 bp. Most of the 10,000 bp regions of scaffold 315 had CQ values above 2 (346/455, 76%), whereas only 2.3% of all the 10,000 bp regions of the genome were found to have a CQ above 2. This result is consistent with this scaffold being part of the X chromosome and visualizations of the male and female coverages across the chromosomal-scale scaffolds (Figure 2) clearly show a lower male coverage for scaffold 315.

There are 260 predicted genes on scaffold 315.^12^ Sixty-one of these are orthologs of *Drosophila* genes and two genes are similar to novel genes in *L. cuprina*. The remaining annotated genes on scaffold 315 are largely repetitive sequences or encode short simple sequence proteins and are not further considered here. For example, g21200 encodes a retrovirus-related Gag protein and g11953 is very similar to several other uncharacterized genes on sc315 and on the autosomal scaffolds. We previously used a copy number approach to identify potential X-linked regions in the *L. cuprina* genome assembly.^41^ We found that orthologs (Reciprocal Best Hits BLAST searches) of 49 of the 59 predicted X-linked genes in *L. cuprina* (83%) are on scaffold 315 (Supplementary Table S5). There are 13 additional protein coding genes present on scaffold 315 that were not predicted to be X-linked in *L. cuprina* (Supplementary Table S5). As six of these genes are on chromosome 4 of *D. melanogaster*, it would seem likely that these additional genes are on the *C. hominivorax* X chromosome, but this remains to be confirmed. The orthologs of three genes that were predicted to be X-linked in *L. cuprina*, *CG33521*, *CG4570*, and *RhoGAP102A* are missing from the *C. hominivorax* assembly. Six of the genes predicted to be X-linked in *L. cuprina* are not present on scaffold 315 but are on one of the large autosomal scaffolds. These are the orthologs of the *D. melanogaster ND-49 (CG1970)*, *CG2022*, *Rho-5*, *Ets65a*, *Rab40*, and *wnk* genes. This could suggest that these genes are on different chromosomes in *L. cuprina* and *C. hominivorax*. However, it is possible that these genes were mis-identified as X-linked in *L. cuprina* since only one of the genes is on the fourth chromosome in *D. melanogaster* (*ND-49*). It is also possible that some regions of the X chromosome have been mis-assembled onto other scaffolds in the current *C. hominivorax* genome assembly. There are two orthologs to the *ND-49* gene in the *L. cuprina* and *L. sericata* genomes that encode proteins that are 90% identical.^42,43^ One gene is X-linked and one is autosomal. Thus, one explanation for the absence of an *ND-49* ortholog from sc315 is that only the autosomal *ND-49* gene has been retained in the *C. hominivorax* genome. However, the protein encoded by the *C. hominivorax ND-49* gene (g18109) shows higher identity to the protein from the *L. cuprina* X-linked *ND-49* gene (96% versus 84% identity). All in all, there appears to be strong conservation of X-linked genes in the two calliphorid species *L. cuprina* and *C. hominivorax.* This analysis will facilitate future studies on the mechanism of X chromosome dosage compensation in these species.

### X- and Y-linked material in unplaced scaffolds

**Figure 3.**
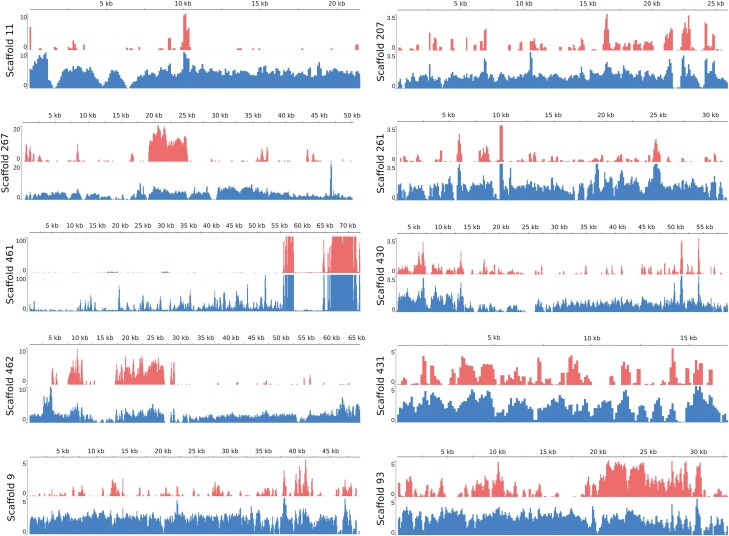
Coverage of female (red) and male (blue) reads across the unplaced scaffolds containing potential Y chromosome regions.

**Figure 4.**
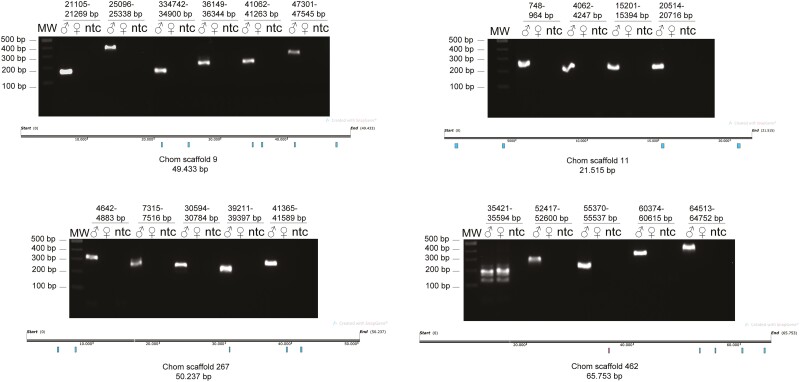
Identification of putative Y-linked fragments in *Cochliomyia hominivorax* scaffolds. NWS male and female adult genomic DNA was used as a template to amplify small portions of Y-linked scaffolds identified by the CQ analysis. PCR products were resolved in 2% agarose gels. Male specificity was defined as the presence of a clear amplicon of a distinct size in male but not in female gDNA samples. A map of each of the analyzed scaffolds shows the regions amplified (cyan male, magenta both sexes). ♂ (male), ♀ (female), ntc (no-template control).

Using the CQ metric, it was also possible to identify possible X and Y chromosome regions in the unplaced scaffolds, which are smaller scaffolds, not incorporated into one of the six large chromosomal scale scaffolds (see Figure 3, Table 3, Supplementary Table S6). We validated a few of the potential Y regions by PCR (Figure 4). From four potential Y scaffolds analyzed (SC9, SC11, SC267, SC462), three were identified as male specific: SC9, SC11 and SC267 (Figure 4, Supplementary Table S1). Interestingly, scaffold SC462 showed only partial Y-linkage, having an estimated ~50% of its sequence Y-linked (Figure 4). This PCR analysis could be used to determine the sex of screwworm at any stage of development, which could be useful for monitoring, for example, future field releases of genetically modified strains in a region with endemic screwworm.

**Table 3.** Unplaced scaffolds with potential Y chromosome regions

Possible linkage	Scaffold	Average scaffold CQ	Genes with congruent CQ
Y	11^*^	0.03	
Y	207	0.5	
Y	261	0.2	
Y	267^*^	0.1	
Y	430	0.3	
Y	431	0.3	g17083 (0.1)
Y	461	0.2	
Y	462^**^	0.3	g10424 (0.0)
Y	9^*^	0.1	
Y	93	0.4	g19341* (0.2)

^*^Confirmed as Y by PCR.

^**^Confirmed as Y by PCR from position 52417-onwards.

The proportion of repeats in the potential sex chromosomes was higher than that of autosomes (for which repeats represents 44.29% of the known bases) with 50.56% and 64.22% of repetitive material on the X chromosome material (scaffold 315 and putative unplaced X chromosome scaffolds listed in Supplementary Table S6) and on the putative Y chromosome scaffolds (Table 3), respectively (Supplementary Table S3). Both the X and the Y material seemed enriched in LTR retrotransposons, which represent 19.5%, 25.7% and 47.4% of the putative autosomal, X and Y material, respectively.

### Identification of Y-linked material in chromosomal-scale scaffolds

**Figure 5.**
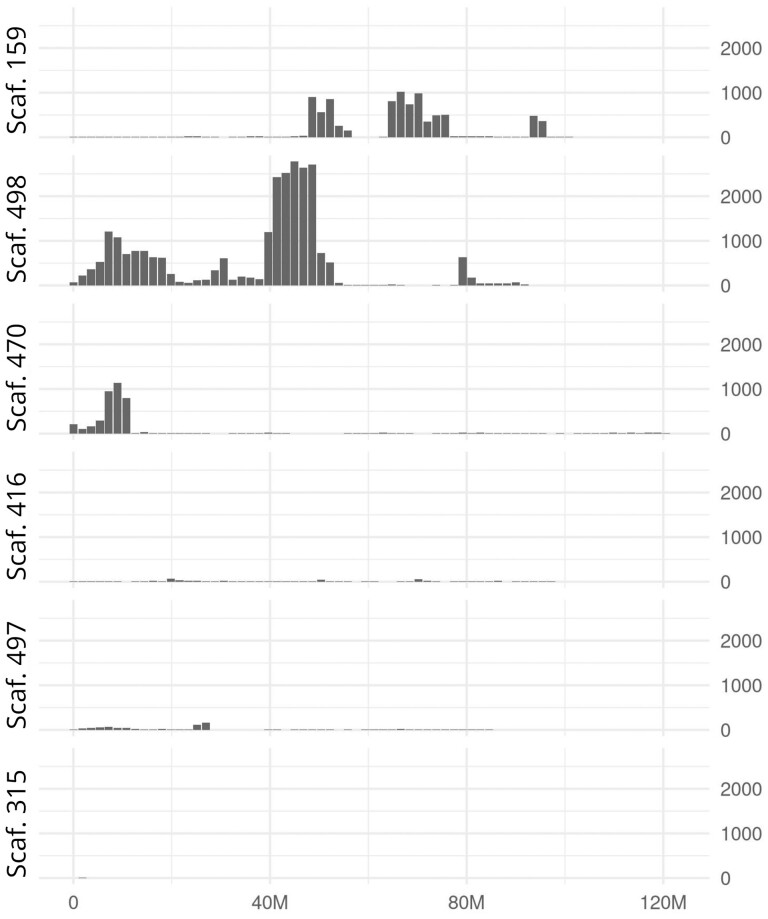
Density of Y-unique regions along the chromosomal-scale scaffolds (in non-overlapping windows of 1.8 Mb). Y-unique regions are continuous regions of 25 bp or more only mapped to by male derived WGS.

**Figure 6.**
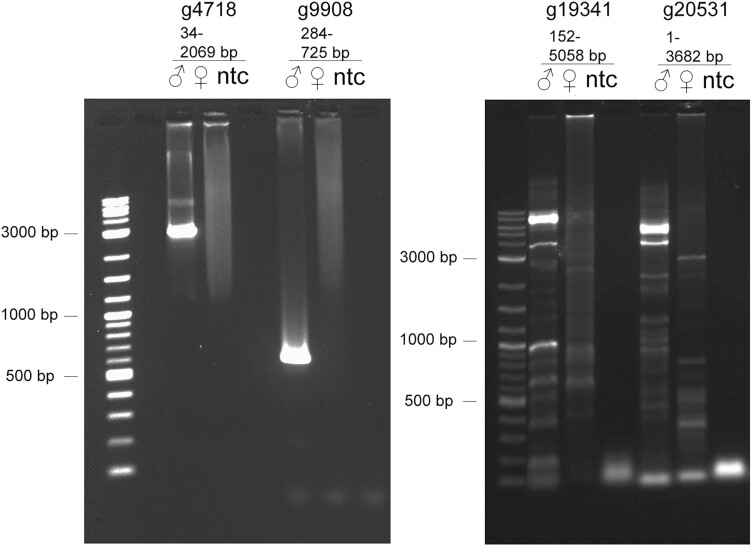
Identification of putative Y-linked genes in *Cochliomyia hominivorax* scaffolds. NWS male and female adult genomic DNA was used as a template to amplify putative Y-linked genes, by long fragment PCR, identified by the CQ analysis. Male specificity was defined as the presence of a clear amplicon of a distinct size in male but not in female gDNA samples.

We also performed a fine-scale analysis to find Y-unique 25bp kmers (25bp sequences only found in the male WGS library) to detect potential Y-linked material in the chromosomal-scale scaffolds. This analysis revealed that some regions of the chromosomal scale scaffolds 498, 159 and 470 showed higher density of Y-unique regions (Figure 5). Amplification of a few genes in these regions with lowish gene and context CQ (<0.6) confirmed them to be Y-linked (Figure 6, Supplementary Table S2). These Y-linked genes are located on small scaffolds in the previous NWS assembly (PRJNA438970) and are usually the only gene on the scaffold. This suggests that the HiC assembly included material from the Y chromosome in the long chromosome-scale scaffolds. The extent of these mis-assemblies is difficult to evaluate but we expect that it is minimal due to the conserved gene content between the chromosomal-scale scaffolds of the current NWS assembly and the chromosomal arms of *D. melanogaster* (Figure 1, bottom line). The reason for these anomalies has yet to be determined but could be due to repeats or interacting regions between distinct chromosomes.

The identification of Y-linked scaffolds will be a valuable resource in the search for the Y-linked male determining gene,^44^ for development of Y-linked Cas9 strains for efficient genetic control of screwworm^45^ and for developing a simple PCR-based assay for sexing screwworm at any stage of development. The observation that some of the chromosome-scale scaffolds appear to include genetic material from the Y-chromosome indicates a potential limitation with HiC genome assemblies, particularly for genomes with highly repetitive sex chromosomes.

In conclusion, the latest genome of *C. hominivorax* presented here is a near-complete chromosome-scale assembly with associated annotation of repeats and coding genes. The genome will facilitate comparative studies among blowflies, and population genomics analyses of screwworm across their current distribution by providing a more global genomic context. This highly continuous version of the genome will be an important resource to locate, in a chromosomal context, markers associated with certain phenotypes, such as parasitism or insecticide resistance. The location of phenotype-related markers or genes is particularly useful to study possible patterns of clusterization or to infer linkage disequilibrium. The identification of X and Y-linked scaffolds will facilitate future studies of X chromosome dosage compensation and Y-linked gene(s) that determine sex. Finally, near chromosome assemblies enable researchers to study the evolution of genome architecture. In 1940, Muller proposed that the genic content of *Drosophila* chromosome arms was conserved within this genus.^46^ With more chromosome-scale genomes of *Diptera* it is possible to extend this concept and infer ancestral karyotypes and gene flows between chromosomes. 

## Supplementary Material

dsac042_suppl_Supplementary_Figure_S1

dsac042_suppl_Supplementary_Tables
