# Supplementary material for: A chromosomal-scale reference genome of the New World Screwworm, Cochliomyia hominivorax
Source: DNA Res. 2022 Nov 12;30(1):dsac042. doi: 10.1093/dnares/dsac042 (PMC9835758; doi:10.1093/dnares/dsac042)
Supplement: dsac042_suppl_Supplementary_Figure_S1 [file dsac042_suppl_supplementary_figure_s1.pdf]

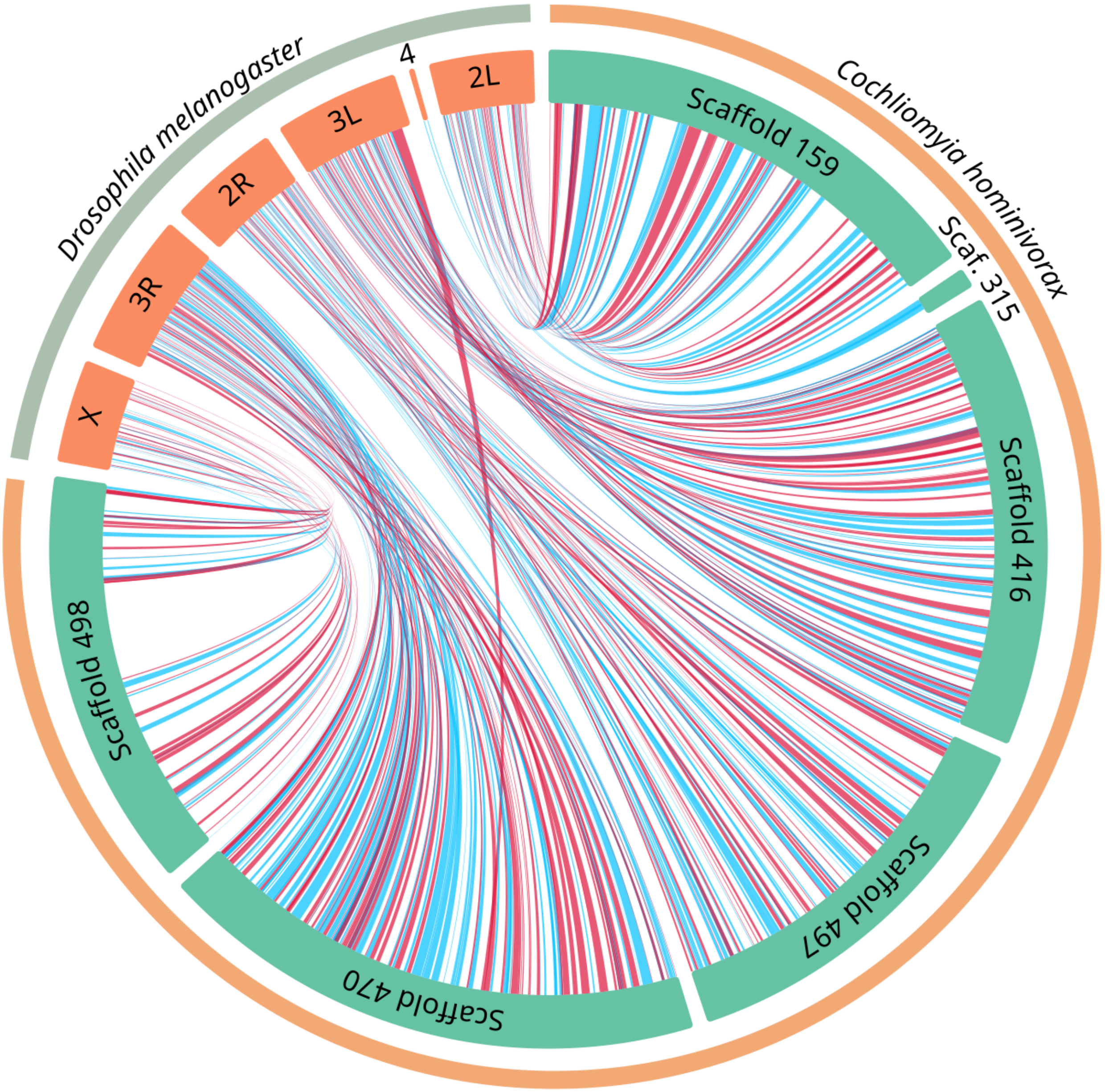

Supplementary Figure S1. Plot depicting the 420 blocks of collinear genes between *D. melanogaster* and *C. hominivorax.* Blocks in the same orientation between *D. melanogaster* and *C. hominivorax* sequences are colored in blue, while blocks of colinear genes with an inverted orientation between both species (flipped blocks) are depicted in red. Only one block was found between non-corresponding *D. melanogaster* chromosomes and *C. hominivorax* scaffolds.
