# Supplementary material for: A chromosomal-scale reference genome of the New World Screwworm, Cochliomyia hominivorax
Source: DNA Res. 2022 Nov 12;30(1):dsac042. doi: 10.1093/dnares/dsac042 (PMC9835758; doi:10.1093/dnares/dsac042)
Supplement: dsac042_suppl_Supplementary_Tables [file dsac042_suppl_supplementary_tables.docx]

A chromosomal-scale reference genome of the New World Screwworm, *Cochliomyia hominivorax*

# Supplementary Material

Sophie Tandonnet¹, Flavia Krsticevic², Tatiana Basika^3^, Philippos Aris Papathanos², Tatiana T. Torres¹, Maxwell J. Scott³

Addresses

¹ Departamento de Genética e Biologia Evolutiva, Instituto de Biociências, Universidade de São Paulo (USP), São Paulo, SP 05508-090, Brazil

² Department of Entomology, Robert H. Smith Faculty of Agriculture, Food and Environment, Hebrew University of Jerusalem, Rehovot 7610001, Israel

³ Department of Entomology and Plant Pathology, North Carolina State University, Campus Box 7613, Raleigh, NC, 27695-7613 USA

ORCIDs

Sophie Tandonnet: 0000-0001-7559-0154

Flavia Krsticevic: 0000-0002-3447-0778

Tatiana Basika: 0000-0003-0823-0591

Philippos Papathanos: 0000-0001-8508-4554

Tatiana T. Torres: 0000-0002-4286-3504

Maxwell J. Scott: 0000-0001-6536-4735

Corresponding author: Maxwell J. Scott

Department of Entomology and Plant Pathology, North Carolina State University, Campus Box 7613, Raleigh, NC, 27695-7613 USA

Tel: 1-919-515-0275, Fax: 919-515-7716

**Supplementary Table S1. Primer sequences of putative Y-linked scaffolds.**

| Scaffold | Forward Primer | Binding site (bp) | Reverse Primer | Binding site (bp) | PCR result |
| --- | --- | --- | --- | --- | --- |
| 9 | AAAGGAGTCCACGAGCAAGA | 21,105 - 21,124 | ACACAGTTTGTTCGCACGAG | 21,250 - 21,269 | only male |
| 9 | TACACCACATCGGGCAGTTA | 25,096 - 25,115 | GCCAAACAAACTTCCTTCCA | 25,319 - 25,338 | only male |
| 9 | TGTTGTGAGCTCCCATTTGA | 34,742 - 34,761 | CTGGCATGAGGAAAGGGTAG | 34,881 - 34,900 | only male |
| 9 | cCCTTGCGGGAATATGGTTTA | 36,149 - 36,169 | AAAATCATCGTGCCGAACTC | 36,325 - 36,344 | only male |
| 9 | CCCATTTTGCTTTGCAATTT | 41,062 - 41,081 | CATGAGCAGCAAGCAGATGT | 41,244 - 41,263 | only male |
| 9 | CACCGCTGAAAAACAGATGA | 47,301 - 47,320 | GGTCATGCGAAGGGACTTTA | 47,526 - 47,545 | only male |
| 11 | TTTTTGCACATTGCGTTTTG | 748 - 767 | TTCCGCATCTTTTCTTTTTCA | 944 - 964 | only male |
| 11 | AATTTCGTGGGGTGGTTTTT | 4062 - 4081 | AGGGAAAATGGGGTTGTGTT | 4228 - 4247 | only male |
| 11 | GCGGAAATATTGTTCAATCCA | 15,201 - 15,221 | TCGACATTACCTTAAAAGCAAAAA | 15,371 - 15,394 | only male |
| 11 | GGTCGCGTAGGACACAGAA | 20,514 - 20,532 | GGGCTGTTCCGGGACTAT | 20,699 - 20,716 | only male |
| 267 | TGCGTCAAGGCACAAAGTTA | 4642 - 4661 | ATTCGATTGTGCCGTTCTTC | 4864 - 4883 | only male |
| 267 | TTTTACGTGCTCCACCATTG | 7315 - 7334 | TGGGAGCTGGGTCAGTTAGT | 7497 - 7516 | only male |
| 267 | CATTGCAATTCCTTCCGAAT | 30,594 - 30,613 | ATTTTGCTTCCGGCCTAGAT | 30,765 - 30,784 | only male |
| 267 | CTGCCAAACCACTTCCACTT | 39,211 - 39,230 | ACGATTGCAGTGCGTTACAC | 39,378 - 39,397 | only male |
| 267 | GTTCCCGGGAGGATCTTTTA | 41,365 - 41,384 | TGATCGGTACGCAATTCTGA | 41,570 - 41,589 | only male |
| 462 | GAGCTTTAGCCCGTGACTTG | 35,421 - 35,440 | TCCGAAAAGCGCTCAAGTAT | 35,575 - 35,594 | both sexes |
| 462 | TCATCATGACCATCCTCAACA | 52,417 - 52,437 | TGCATACACGTGGAACACAA | 52,581 - 52,600 | only male |
| 462 | TGGACATTGCGTTTGTGTTT | 55,370 - 55,389 | ACGGTATGGGTGAGTGCTTC | 55,518 - 55,537 | only male |
| 462 | TCAAGGTCTCCCACCAAAAC | 60,374 - 60,393 | TGTCTCTCTGGCTGCCTGTA | 60,596 - 60,615 | only male |
| 462 | GGATTGAAACGTGCAAGGAT | 64,513 - 64,532 | TTACCGACTGTGCTCATGGA | 64,733 - 64,752 | only male |

**Supplementary Table S2. Primers for Y-linked genes**

| Gene | Forward Primer | Binding site (bp) | Reverse Primer | Binding site (bp) | PCR result |
| --- | --- | --- | --- | --- | --- |
| g4718 | TGCACAATTCTCACGACCTC | 34 - 53 | ACCGTCCTGACCAAAAGATG | 2050 - 2069 | only male |
| g9908 | AATGCGTCATTTGGTCACAA | 284 - 303 | TCCCTCAGCCAATTTATGGT | 706 - 725 | only male |
| g19341 | GTATTTTCGCGGTTTTTGGA | 152 - 171 | TTGCAATCGAACCCAAATTA | 5039 - 5058 | only male |
| g20531 | ATGCTGAATGATGCTTCTGCTGCC | 1 - 24 | TAAATGGTGGCAATCGTGAA | 3663 - 3682 | only male |

**Supplementary Table S3. Statistics of each of the chromosomal-scale scaffolds and putative sex chromosome material of *C. hominivorax*.** *‘*Dmel’ and ‘Lcup’ stand for ‘*Drosophila melanogaster*’ and ‘*Lucilia cuprina*’, respectively.

| Chromosomal Scaffold | Corres-pondence to *Dmel* | Corres-pondence to *Lcup* | Length (bp) | Number of genes | Repeat length (bp) (%***) | Number of gaps | %GC |
| --- | --- | --- | --- | --- | --- | --- | --- |
| Scaffold_498 | X | Chr3 | 93.88 | 3,907 | 37762220 (40.25%) | 721 | 27.72 |
| Scaffold_159 | 2L | Chr2 | 103.54 | 3,801 | 46,035,207 (44.50%) | 915 | 27.90 |
| Scaffold_497 | 2R | Chr6 | 89.25 | 3,838 | 41,681,238 (46.73%) | 598 | 27.75 |
| Scaffold_416 | 3L | Chr5 | 101.52 | 3,947 | 45157867 (44.52%) | 827 | 27.85 |
| Scaffold_470 | 3R | Chr4 | 123.43 | 4,602 | 52714484 (42.73%) | 800 | 27.53 |
| Scaffold_315 | 4 | ChrX | 4.56 | 260 | 2,269,572 (49.77%) | 27 | 28.79 |
| Potential X scaffolds* + Scaffold_315 | NA | NA | 4.97 | 281 | 2,513,645 (50.56%) | 30 | 28.54 |
| Potential Y Scaffolds** | NA | NA | 0.41 | 17 | 264459 (64.22%) | 5 | 26.75 |

* Total metrics from Scaffold_315 as well as from the 16 potential X chromosome scaffolds listed in Supplementary Table S5

** Total metrics from the 10 potential Y scaffolds listed in Table 3

*** The calculation of the percentage of repeats did not consider runs of Ns.

**Supplementary Table S4. Collinear blocks between *C. hominivorax* and *D. melanogaster.*** The notation 'Chom' and 'Dmel' stand for *C. hominivorax* and *D. melanogaster*, respectively.

| Chromosomal pair | # collinear blocks (same orientation) | # collinear blocks (flipped orientation) |
| --- | --- | --- |
| Chom_Scaffold_159 & Dmel_2L | 39 | 33 |
| Chom_Scaffold_498 & Dmel_X | 18 | 21 |
| Chom_Scaffold_470 & Dmel_3R | 69 | 62 |
| Chom_Scaffold_416 & Dmel_3L | 47 | 45 |
| Chom_Scaffold_497 & Dmel_2R | 46 | 37 |
| Chom_Scaffold_315 & Dmel_4 | 2 | 0 |
| Chom_Scaffold_470 & Dmel_3L | 0 | 1 |

**Supplementary Table S5. Predicted genes on the X chromosome scaffold 315.**

| *Cochliomyia hominivorax* gene ID | *Drosophila melanogaster* ortholog | *Drosophila melanogaster* linkage | Predicted to be X-linked in *L. cuprina* |
| --- | --- | --- | --- |
| g406 | *Ank* | 4 | Yes |
| g409 | *Cals* | 4 | Yes |
| g14322 | *Sox102F* | 4 | Yes |
| g11116 | *eIF4G* | 4 | Yes |
| g11122 | *fd102C (CG11152)* | 4 | Yes |
| g11098 | *lgs* | 4 | Yes |
| g11105 | *CG1814* | 2R | Yes |
| g11092 | *CG1909* | 4 | Yes* |
| g11097 | *mav* | 4 | Yes* |
| g21187 | *CG1674* | 4 | Yes |
| g21204 | *PMCA (CG42314)* | 4 | Yes |
| g11952, g11954 | *CG31998* | 4 | Yes |
| g21197 | *zfh2* | 4 | Yes* |
| g11963 | *MED26* | 4 | Yes |
| g21190 | *4E-T (CG32016)* | 4 | Yes |
| g5006 | *sv* | 4 | Yes |
| g9163 | *ey* | 4 | Yes |
| g394 | *Arl4 (CG2219)* | 4 | Yes |
| g419 | *yellow-h* | 4 | Yes* |
| g423 | *Kif3C* | 4 | Yes |
| g390 | *pho* | 4 | Yes |
| g415 | *Eph* | 4 | Yes* |
| g11095 | *gw* | 4 | Yes* |
| g7676 | *aru* | 2L | Yes* |
| g7679 | *Jwa* | 2L | Yes* |
| g11088 | *CG2316* | 4 | Yes |
| g11089 | *CG31999* | 4 | Yes |
| g11962 | *PlexA* | 4 | Yes |
| g11960 | *fuss (CG11093)* | 4 | Yes |
| g21188 | *Asator* | 4 | Yes |
| g11094 | *Crk* | 4 | Yes |
| g9150 | *PlexB* | 4 | Yes |
| g21210 | *CG11360* | 4 | Yes |
| g21199 | *Actbeta* | 4 | Yes |
| g11117 | *CG33978* | 4 | Yes |
| g9159 | *Thd1* | 4 | Yes* |
| g9142 | *dpr7* | 4 | Yes |
| g11111 | *CG31997* | 4 | Yes |
| g11112 | *unc-13* | 4 | Yes |
| g21202 | *Slip1* | 4 | Yes* |
| g21211 | *CG32850* | 4 | Yes |
| g21189 | *CG32006* | 4 | Yes |
| g21195 | *ci* | 4 | Yes |
| g11967 | *toy* | 4 | Yes |
| g411 | *pan* | 4 | Yes |
| g9145 | *Lin29 (CG2052 or dati)* | 4 | Yes |
| g10870 | *myo (CG1838)* | 4 | Yes |
| g7649 | *bt (bent)* | 4 | Yes |
| g21191 | *onecut* | 4 | Yes |
| g11124 | *FoxP* | 3R | No |
| 18109 | *Lcup FF38_09679* | N/A | No |
| g11123 | *Lcup FF38_11593* | N/A | No |
| g11946 | *Ca-alpha1D* | 2L | No |
| g11949 | *PIP4K* | 4 | No |
| g12418 | *CG5367* | 2L | No |
| g21198 | *apolpp* | 4 | No |
| g7660 | Gigyf  *(CG11148)* | 4 | No |
| g9157 | *MFS17* | 2R | No |
| g7665 g7664 | *CG4972 (*Nicalin) | 2L | No |
| g21212 | *CG11155* | 4 | No |
| g416 | *Arf102F* | 4 | No |
| g11091 | *Gat (CG1732)* | 4 | No |

*confirmed by qPCR

**Supplementary Table S6. Unplaced scaffolds with potential X chromosome regions.**

| Possible linkage | Scaffold | average scaffold CQ | genes with congruent CQ |
| --- | --- | --- | --- |
| X | Scaffold 114 | 16.8 | g20224 (3.0) |
| X | Scaffold 123 | 3.1 |  |
| X | Scaffold 136 | 2.9 | g21090 (2.7), g21092 (3.8) |
| X | Scaffold 138 | 3.9 |  |
| X | Scaffold 194 | 3.1 | g3740 (2.07) |
| X | Scaffold 223 | 2.2 |  |
| X | Scaffold 225 | 2.8 |  |
| X | 312 | 2.2 |  |
| X | 316 | 5.5 |  |
| X | 427 | 2.3 | g16595 (25.1), g16596 (4.7) |
| X | 473 | 2.1 |  |
| X | 50 | 2.3 |  |
| X | 516 | 2.7 |  |
| X | 521 | 2.1 |  |
| X | 524 | 2.1 |  |
| X | 63 | 1.9 | g18459 (4.5), g18460 (5.9) |
